# Characterization of SSR markers from draft genome assembly and genotypic data in *Hedychium spicatum* (Zingiberaceae)

**DOI:** 10.1016/j.dib.2024.110568

**Published:** 2024-05-29

**Authors:** Aleena Xavier, Vinita Gowda

**Affiliations:** Tropical Ecology and Evolution (TrEE) Lab, Department of Biological Sciences, Indian Institute of Science Education and Research Bhopal (IISER Bhopal), Madhya Pradesh 462066, India

**Keywords:** Allelic diversity, Asian tropics, Microsatellites, Fragment analysis, Genotyping, North East India, Rhizomatous herb, Spiked ginger lily

## Abstract

The plant family Zingiberaceae consists of many medicinally important tropical herbs. Here, we provide a contig level genome assembly for *Hedychium spicatum*, one of the medicinally utilized species in this family*.* We used genome assembly to identify candidate Simple Sequence Repeat (SSR) markers in the nuclear, chloroplast and mitochondrial compartments. We identified a total of 60,695 SSRs, which consisted of di-, tri-, tetra-, penta- and complex repeat types, and primers were designed for 14,851 SSR loci from both coding and non-coding parts of the genome. A total of 62 sets of candidate SSR primers were tested, out of which a final set of 20 SSR markers were characterized and they met the criteria of amplification success and retention of the repeat motif and homology. Out of these 20 markers, we genotyped 11 markers by amplifying and sizing 99 accessions of *H. spicatum* from 13 different geographic locations. The 11 markers were also characterised for four congeneric species, *H. ellipticum, H. gomezianum, H. venustum*, and *H. yunnanense*. All 11 SSR markers were found to be polymorphic and showed cross-species amplification. The total number of alleles per locus varied from 5 to 25. SSR markers continue to be a valuable tool for researchers because of their cost-effectiveness and simplicity. The cross-species amplification and variability of the SSR markers generated here further extend the utility of the markers to other *Hedychium* spp. The markers presented in this dataset can be used for a variety of studies, such as population genetics of invasive *Hedychium* species, QTL mapping, DNA fingerprinting, parentage analysis and genetic diversity assessments.

Specifications Table

**Table:** 

Subject	Biological Sciences
Specific subject area	Plant genomics, Bioinformatics, Biodiversity, Molecular biology, Phylogeny and Evolution, Systematics, Ecology and Behaviour
Type of data	Tables, Figures Raw, Filtered, Analysed, and Processed
Data collection	Genomic DNA was extracted for *Hedychium* species using Qiagen DNeasy® plant mini kit, followed by whole genome sequencing on the Illumina HiSeq platform using the NEB NEXT DNA II Library prep kit. MISA was used to find candidate SSR loci from the genome assembly and primers were designed using Primer3. After PCR amplification, candidate SSR fragments were sized on Agilent 2100 Bioanalyzer with Agilent DNA 1000 Kit and sequenced on Applied Biosystems 3500 genetic analyzer. Test individuals from five *Hedychium* species were genotyped using fluorescently labelled SSR primers tagged with FAM™, VIC™, NED™, or PET™, and with GeneScan™ 600 LIZ™ as size standard on the Applied Biosystems 3500 genetic analyzer.
Data source location	Collection source: India, Nepal, Bhutan and China (geographical coordinates uploaded to the repository [1]) Institution: All accessions used in this study are deposited in BHPL herbarium, IISER Bhopal, India.
Data accessibility	Repository name: Mendeley Data Data identification number: /10.17632/zm79tkwnbc.1 [1] Direct URL to data: https://data.mendeley.com/datasets/zm79tkwnbc/1

## 1 Value of the Data

• The draft genome provided here for *Hedychium spicatum* increases the genetic information available for the medicinally important gingers (family Zingiberaceae).
• The 14,851 candidate SSR markers, along with their annotations, provide a comprehensive mapping of these markers across the genome of a non-model organism.
• The 11 variable SSR markers identified using characterization in five *Hedychium* species can be utilized for genetic diversity assessment, QTL mapping, and to differentiate between different accessions of horticulturally and medicinally important species of *Hedychium spicatum* and allied species.

## 2 Background

Simple Sequence repeat (SSR) markers were developed as part of a systematic study of the ginger genus *Hedychium,* which contains many species complexes [2]. Delimiting species within species complexes requires molecular markers that can show individual-level variations, and SSRs are ideal markers due to their codominant nature, robustness, ease of use, and cost-effectiveness [3]. Population genetic studies in gingers (Zingiberaceae) have been mostly restricted to commercially important species; therefore, SSR markers were available only for *Curcuma longa* (Turmeric [[4], [5], [6]]), *Zingiber officinale* (Ginger [[7], [8], [9]]), and *Elettaria cardamomum* (Cardamom [10]), all of which are distantly related to the genus *Hedychium*. During an initial inter-generic amplification and screening of the SSR markers (total *n* = 42; Details uploaded to [1]) that were developed for the above-mentioned three ginger species, only ten markers were amplified in *H. spicatum* and only one showed the presence of the repeat motif. This indicated that the intergeneric cross-compatibility of the available SSR markers was limited, thereby restricting their application in *Hedychium*. As a result, we developed new SSR markers for *Hedychium* by using shallow genome sequencing and characterized a few of them, which can be utilized for calculating inbreeding and outcrossing rates and inferring the population genetic and phylogeographic patterns.

## 3 Data Description

**Fig. 1:**
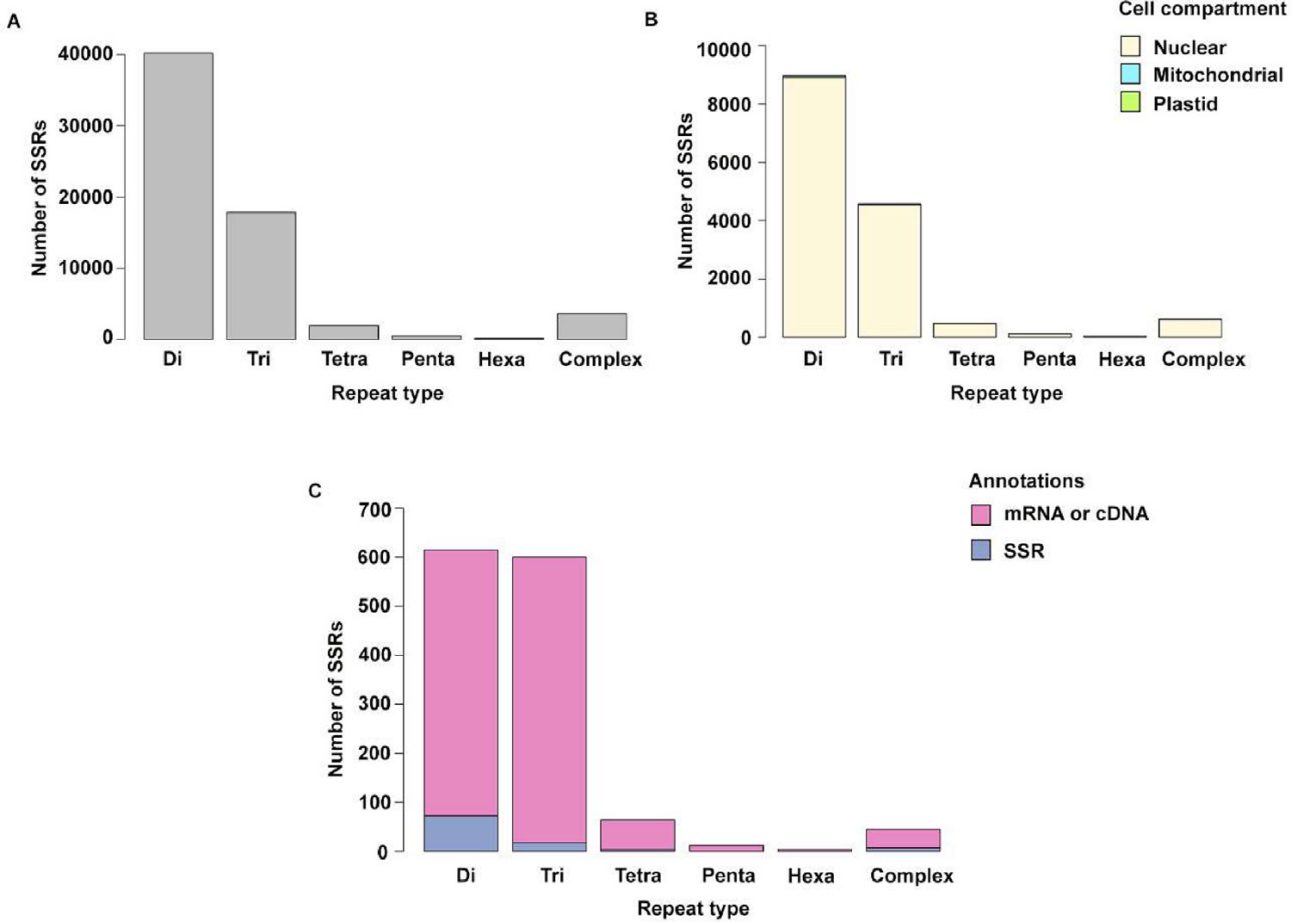
Distribution of the different SSR markers in *H. spicatum*. A, the total number of SSRs identified in this study and their repeat type; B, the total number of SSRs for which primers were designed and their assigned cell compartment and repeat type; C, the total number of SSRs annotated and identified to be either from mRNA or cDNA and SSRs.

The whole genome sequence of *H. spicatum* assembled at the contig level can be found in the Mendeley data repository (https://data.mendeley.com/datasets/zm79tkwnbc/1), and it is also uploaded to NCBI (Accession no: JBBJBY000000000, BioProject: PRJNA1087568). The assembly has a total length of 566.3 Mb consisting of 196,163 contigs, with 20,784 contigs having lengths above 1000 bp, and the longest contig was 6794 bp long. From this assembly, MISA [11] identified a total of 60,695 SSRs (Fig. 1A), and primers were designed for 14,851 SSR loci (data available at: https://data.mendeley.com/datasets/zm79tkwnbc/1), out of which 138 loci were annotated as part of plastome or mitochondrial DNA (Fig. 1B). Out of the 14,713 nuclear SSR loci, 1208 loci were annotated to be either part of a mRNA or protein coding region and only 100 loci were annotated as SSR loci (Fig. 1C). Among the SSRs annotated to be a part of mRNA, the most common repeat motifs were tri repeats (583) followed by di repeats (543). The most common repeat motifs in the genome were di repeat, followed by tri-, compound-, tetra-, penta-, and hexa- repeats (Fig. 1A). The most common repeats consisted of AT repeats followed by GC [1].

Using eight test samples of *H. spicatum* (4 samples each from 2 populations) we finalised 20 markers from the 62 candidate SSR markers (List available at: https://data.mendeley.com/datasets/zm79tkwnbc/1). The final list of 20 markers met all the following three criteria: (i) successful amplification, (ii) presence of the targeted repeat motif and flanking region, (iii) presence of fragment length polymorphism. These 20 primers included five di-repeats, eight tri-repeats, four tetra-repeats, two penta-repeats and one complex repeat (Table 1). The optimal annealing temperature of these primers, along with their fragment size ranges estimated from the Bioanalyzer are given in Table 1.

**Table 1:** Detailed characteristics of 20 SSR markers based on their amplification, sequencing, and fragment size evaluation in eight test samples of *H. spicatum*. Ta- annealing temperature. Labelling of primers shown for the first 11 SSR markers which were used for further genotyping.

Sl no	Marker name	Motif	Repeat type	Size range	Forward primer	Reverse primer	Ta °C	Label used
1	5-GT14	GT	di-	197–223	ACTGTTGATGGGACTGAGCA	GACGGTTCGTGCTGTGAATG	58	6-FAM
2	16-GCAC6	GCAC	tetra-	160–218	CGTTTCCTAATGGCTGCTCG	CTCGTTGTCCCTGATACGCA	60	PET
3	19- ATATA8	ATATA	penta-	213–340	GGTGGCCGGGTGATAGAATC	CCCAAGTGGAAGCCTCACTT	58	VIC
4	27-TG12	TG	di-	118–164	AGGTTCGTCATGGTAAGTGCT	AGTCAGACATTGAAGGCCCT	58	VIC
5	43-AG10	AG	di-	80–118	TGATCGAAGGCTAGCTAAACGA	AACCAACTCTTCCCTCTCGC	60	PET
6	45-GA15	GA	di-	126–220	AGGGACTGTGGATTAAGCACT	ACTGACATGCCACAGAAACA	58	NED
7	20-GAAGG7	GAAGG	penta-	195–215	CCTGCTGCACTCATCCTCTA	TCCTCCAAGTCAGTCCGGAT	60	NED
8	33-GAA18	GAA	tri-	188–284	AACACGTCAGAGGGCTTGTT	TCCCCTTCCAACCTAGCAGA	58	VIC
9	50-CAT9	CAT	tri-	149–179	ACCGAGCTCGAGTCTTTTACT	ACCTTGAGACGAGAACTGGC	60	6-FAM
10	49-AGA9	AGA	tri-	203–224	AGTGTCGCAATGGCTCTGTT	CCTGTACAATGGCTTCGGGA	60	6-FAM
11	54-TTC9	TTC	tri-	195–223	GCGGAGGTGCTCGAATATCA	CCCCTCTAGCTCGCCAAGTA	60	VIC
12	21-TA6TATG6	(TA) (TATG)	complex	169–217	CATCTGAGTTGGCAGTCCTT	TCGACGACAACATTGGTCCA	58	–
13	30-AGT10	AGT	tri-	189–333	TGGATGAAGAGGTGCTTGCT	GGTTCGTCACCTTTCCACCA	60	–
14	35- TTAA6	TTAA	tetra-	133–220	ACCCTACTTAGCTTGTGGTTCC	AGGTCAATTACGTACGTCAACCA	58	–
15	47-TA17	TA	di-	124- 217	TGGATGATGTGCACAAAGTCT	CCTCCTTCCCCATATCAGCC	58	–
16	51-CCT9	CCT	tri-	185–200	AACCCTGGTTGTTGGGATCG	TGGGCTATGCGTTTGGTGAT	58	–
17	52-TAT9	TAT	tri-	195–213	CCAACAGGGCTTAGGGTTGT	AGATCGCTTGACGGAGAAGC	60	–
18	53-TTA13	TTA	tri-	180–220	AGGGTTTAGGGAATCACTGAGT	TCTATGGAACGGGTGGTCCA	58	–
19	15-AATT6	AATT	tetra-	180–248	CATTCCATCCACCAGGCCTT	TTGGAGGACAACGCAGGATA	58	–
20	55-AACA6	AACA	tetra-	165–175	TCAGCCTTCTCAAACCACACT	AGTTTTGCATGATGTCAATTATCAATT	60	–

**Fig. 2:**
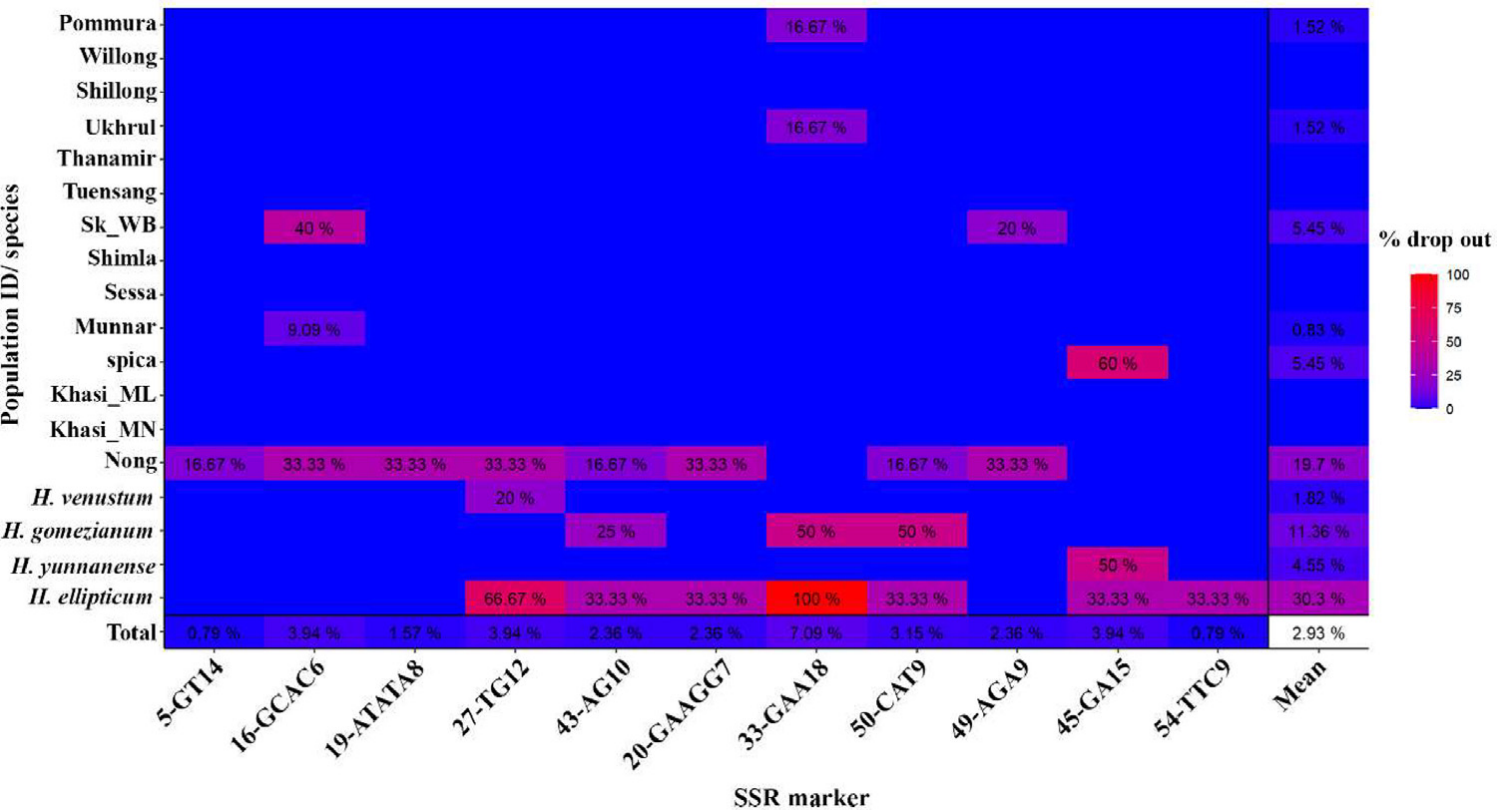
Heat map showing amplification success rates of 11 SSR markers used for genotyping five species of *Hedychium*. Percent success rate is shown in blue (100 %) and percent failure rate is shown in red (100 %). Population from Pommura to Munnar represent different populations of *H. spicatum* from India*,* while spica is *H. spicatum* from Nepal, China and Bhutan, and Khasi_ML and Khasi_MN -Khasianum are two morphological forms of *H. spicatum* from Meghalaya and Manipur in India respectively, and Nong- Nongstoin is a morphological form of *H. spicatum* from Meghalaya, India.

Further detailed genotypic characterization was carried out for 11 out of the 20 finalised markers (Tables 1 and 2) for selected test samples (*n* = 127 individuals [1]) across five species and a total of 13 populations ([1]; also see experimental design for details). Although the 11 SSR markers successfully amplified in all test populations of *H. spicatum*, it showed some variability in amplification success among individuals within a population (Fig. 2). For example, seven out of 11 markers amplified in all test individuals (99) of *H. spicatum* (Fig. 2.) while four markers dropped out in a few individuals from specific *H. spicatum* population and in some morphological variants of *H. spicatum* (Khasianum form and Nongstoin form, Fig. 2). Amplification success was also variable among the four congeneric gingers *H. ellipticum, H. gomezianum, H. venustum, H. yunnanense*, for example, 33-GAA18 did not amplify in *H. ellipticum* (Fig. 2). The amplification success rate (in blue) and failure rate (in red) across all *Hedychium* populations and for all the 11 SSR markers is given in Fig. 2. The mean missing data in the final dataset [1] is 2.93 %, and the highest locus dropout is observed in *H. ellipticum* (30.3 %), followed by the Nongstoin form (19.7 %). The SSR marker with the highest dropout rate is 33-GAA18 (7.09 %; Fig. 2).

**Table 2:** Allelic characterization of 11 SSR markers. H- observed Shannon-Wiener diversity index for the marker, H-exp- expected genetic diversity for the marker.

Marker	No: of alleles	H	H-exp	Evenness
5-GT14	10	0.843	0.847	0.886
16-GCAC6	6	0.671	0.674	0.708
19-ATATA8	25	0.906	0.909	0.722
27-TG12	12	0.865	0.868	0.861
43-AG10	6	0.719	0.722	0.819
20-GAAGG7	5	0.681	0.684	0.911
33-GAA18	9	0.801	0.805	0.846
50-CAT9	13	0.697	0.700	0.565
49-AGA9	10	0.784	0.787	0.754
45-GA15	15	0.692	0.695	0.472
54-TTC9	8	0.787	0.790	0.835
Mean	10.818	0.768	0.771	0.762

The mean number of alleles per loci is ∼11, and the number of alleles ranged from five in 20-GGAGG7 to 25 in 19-ATATA8. The allelic details of each SSR marker, such as the number of alleles observed per locus along with evenness, expected allelic diversity, and observed allelic diversity, are given in Table 2. The Shannon-Weiner diversity varied between different markers and it ranges from 0.67 in 16-GCAC6 to 0.91 in 19-ATATA8. The allelic evenness of the different SSR markers varied from 0.47 (in 45-GA15) to 0.91 (in 20-GAAGG7; Table 2).

## 4 Experimental Design, Materials and Methods

### 4.1 Shallow genome sequencing and assembly

To find the candidate microsatellite markers, we used shallow genome sequencing in *H. spicatum* that was collected from Pommura, Meghalaya, India (collection ID: VG_2018_ML_1792, deposited in BHPL). The genomic DNA extraction was performed using the Qiagen DNeasy® plant mini kit following the standard protocol, except for a change in the incubation time in AP1 lysis buffer, which was increased from 10 min to 1 hr as it was noted to give an improved yield. The gDNA extracted was sent to Clevergene Biocorp Pvt. Ltd, Bengaluru, for library preparation and sequencing. For library preparation 1ug of genomic DNA was fragmented using an ultrasonicator (Covaris M220) to generate a mean fragment size of 350 bp. The NEB NEXT DNA II Library prep kit for Illumina was used to make the genomic library. Library quantity and quality were then checked using Qubit HS dsDNA assay and Agilent Bioanalyzer DNA HS assays. The samples were then sequenced on the Illumina HiSeq platform to generate 2 × 150 bp reads. The base call files were converted to FASTQ files using the bcl2fastq program. The quality of the sequence data was checked using FastQC [12] and MultiQC software [13]. The data was then processed to remove adapter sequences and low-quality reads using fastp [14]. The processed reads were assembled using ABySS v2.0.2 [15]. Four k-mer sizes of 49, 67, 99 and 121, were used to assemble the reads. Contigs shorter than 200 bp were removed from the assemblies. The assembled genomes were compared using QUAST [16]. To assess the assembly quality, trimmed reads were mapped back to the assembled genomes using Bowtie2 [17]. The assembly generated with k-mer size 67 was 566.3 Mb in size, and this assembly was most informative since it had the highest number of reads (75.41 % of reads) being mapped back. Therefore, this assembly was considered for SSR discovery. The contigs were annotated by BLAST against the NCBI nucleotide database with default parameters [1].

### 4.2 Identification of SSR markers

The short sequence repeats (SSRs) were identified using MISA [11], where repeat motifs of 2–6 bases with at least six repeats for the dinucleotide repeat motif and five repeats for the rest of the repeat motif were identified. If two or more SSRs were within a 100 bp region, they were considered ‘complex SSRs’. From all the SSRs identified by MISA, primers were designed for markers that had at least 100 bp of the non-repetitive flanking region, using the default parameters in Primer3.

From 14,851 candidate SSR markers, 62 markers were shortlisted (Table uploaded to Mendeley database), which were not found to occur in a coding region. The SSR loci in the coding region were avoided as they might be under strong selection for repeat length, resulting in the absence of repeat length variation. Further, we made sure that all repeat types (di-, tri-, tetra-, penta- and hexa-) were included in the shortlisted set of final markers to capture variable mutation rates from the different repeat types.

The forward and reverse primers for the 62 markers had a melting temperature ranging from 58 to 60 °C. These 62 primers were first amplified in the test sample (collection no: VG_2018_ML_1792, deposited in BHPL) that was used for the shallow genome sequencing. 10 µl PCR reaction was set up for each primer pair with 0.5 µl of 10uM forward primer and reverse primer each, 0.2 µl of Taq polymerase (Bioline MyTaq™ Polymerase), 2 µl of 5x reaction buffer, 1 µl of template DNA and 5.8 µl of nuclease-free water. We used a touchdown PCR protocol which had an initial denaturation for 3 min at 95℃, ten cycles of denaturation at 95℃ for 15 s, annealing at 60℃ for 20 s, extension at 72℃ for 30 s, followed by ten cycles with annealing temperature at 58℃ followed by ten cycles with annealing temperature at 56℃, and a final extension at 72℃ for 7 min. The PCR products were run on a 3 % MetaPhor™ Agarose gel with ethidium bromide (EtBr) in 1x tris borate EDTA buffer (TBE) for 1 hour at 100v and visualized under UV using UVP GelDoc-It^2^ imaging system. All amplified products with more than two bands on the gel (non-specific amplifications) were discarded, and PCR clean-up was carried out using ExoSAP-IT™. For the clean-up, 5 µl of PCR product and 1 µl of ExoSAP reagent were used, and the following reaction was set up in the PCR machine with 37℃ for 15 min followed by 80℃ for 15 min. Next, these samples were sequenced on the Applied Biosystems genetic analyzer 3500 platform using the Sanger sequencing method. The sequences were examined using Geneious 11.1.5 software to make sure that the target motif was being amplified. Markers with no repeat motifs were discarded, while the markers that had the repeat motif were amplified in eight *H. spicatum* test samples (4 samples per population) from Pommura in Meghalaya, India and Eaglenest in Arunachal Pradesh, India. All these samples were again sequenced using Sanger sequencing to confirm that the target region was being amplified for samples from all the test populations. The fragments were sized in the Agilent 2100 Bioanalyzer using an Agilent DNA 1000 Kit. The markers that showed fragment size polymorphism were then shortlisted to be labelled with the four fluorescent markers, FAM™, VIC™, NED™ or PET™ (see Table 1).

### 4.3 Genotypic characterization of SSR markers

The labelled primers were finally amplified in a total of 127 test samples, which consisted of *H. spicatum* from thirteen populations (1–12 individuals per population), two intermediate forms of *H. spicatum* (*n* = 14), and four congeneric *Hedychium* species (total *n* = 14) - *H. venustum, H. gomezianum, H. ellipticum and H. yunnanense* (Details can be found at: https://data.mendeley.com/datasets/zm79tkwnbc/1). PCR amplification consisted of a 10 µl PCR reaction with 0.3 µl of 10 µM forward and reverse primers each, 0.1 µl of Taq polymerase (Bioline MyTaq™ Polymerase), 2 µl of 5x reaction buffer, 1 µl of template DNA (at 1:5 dilution) and 6 µl of nuclease-free water. The PCR protocol consisted of an initial denaturation step of 3 min at 95℃, 30 cycles of denaturation at 95℃ for 15 s, annealing at 58℃−60℃ (Table 1) for 20 s, and a final extension at 72℃ for 7 min. The PCR products were then checked on a 2 % agarose gel with EtBr in sodium borate (SB) buffer and visualized under the UVP GelDoc-It^2^ imaging system. The PCR products were cleaned up using EXOSAP-IT™, and the samples were then either diluted at 1:10 or 1:20, depending on the intensity of the band in the gel. The samples were then run on the Applied Biosystems Genetic analyzer 3500 for sizing of the fragments. We used a Liz dye system where the size standard is labelled with LIZ™, which allows for multiplexing of four SSR marker. GeneScan™ 600 LIZ™ dye size standard was used as a size standard since it accurately sizes fragments from 20 bp to 600 bp, and all of our expected fragment size ranges were within this range. For each sample, we used 1 µl of the sample, 0.5 µl of size standard and 8.5 µl of HiDi Formamide. A denaturation step was set up in the PCR machine at 94℃ for 3 min, and the samples were loaded on Applied Biosystems Genetic analyzer 3500 for fragment sizing. Fragment sizes were reported in ‘.fsa’ files.

The ‘.fsa’ files from the genetic analyzer were analysed for fragment size variability within and among populations using the MSA (microsatellite analysis) tool in the Thermo Fisher cloud (https://apps.thermofisher.com/apps/). The size standard peaks were checked first to make sure that all the size standard peaks were detected. The peaks of multiple samples were checked to know the size variation for a marker and bins were created based on the variation to automate the sizing. Size information for all the samples across the 11 SSR markers was tabulated using MSA. This table was then used to infer the number of alleles, allelic diversity and the evenness of the alleles for each of the markers using the R package ‘poppr’ [18] in R 4.1.1.

## Limitations

None.

## Ethics Statement

All the authors have read and followed the ethical requirements for publication in Data in Brief and we confirm that the work given here does not involve human subjects, animal experiments or any data collected from social media platforms.

## CRediT authorship contribution statement

**Aleena Xavier:** Conceptualization, Investigation, Formal analysis, Validation, Data curation, Writing – original draft. **Vinita Gowda:** Conceptualization, Investigation, Data curation, Funding acquisition, Writing – review & editing, Supervision, Project administration.

## Data Availability

Dataset of SSR markers for Hedychium spicatum (Zingiberaceae) based on draft genome assembly and experimental characterization of few markers (Original data) (Mendeley Data).
